# TF-LIME : Interpretation Method for Time-Series Models Based on Time–Frequency Features

**DOI:** 10.3390/s25092845

**Published:** 2025-04-30

**Authors:** Jiazhan Wang, Ruifeng Zhang, Qiang Li

**Affiliations:** School of Microelectronics, Tianjin University, Tianjin 300072, China; itswzz8@tju.edu.cn (J.W.); zhangruifeng@tju.edu.cn (R.Z.)

**Keywords:** time series data, explainability, time–frequency domain, LIME, feature attribution methods

## Abstract

With the widespread application of machine learning techniques in time series analysis, the interpretability of models trained on time series data has attracted increasing attention. Most existing explanation methods are based on time-domain features, making it difficult to reveal how complex models focus on time–frequency information. To address this, this paper proposes a time–frequency domain-based time series interpretation method aimed at enhancing the interpretability of models at the time–frequency domain. This method extends the traditional LIME algorithm by combining the ideas of short-time Fourier transform (STFT), inverse STFT, and local interpretable model-agnostic explanations (LIME), and introduces a self-designed TFHS (time–frequency homogeneous segmentation) algorithm. The TFHS algorithm achieves precise homogeneous segmentation of the time–frequency matrix through peak detection and clustering analysis, incorporating the distribution characteristics of signals in both frequency and time dimensions. The experiment verified the effectiveness of the TFHS algorithm on Synthetic Dataset 1 and the effectiveness of the TF-LIME algorithm on Synthetic Dataset 2, and then further evaluated the interpretability performance on the MIT-BIH dataset. The results demonstrate that the proposed method significantly improves the interpretability of time-series models in the time–frequency domain, exhibiting strong generalization capabilities and promising application prospects.

## 1. Introduction

Time series data, common forms of data representation, have been widely applied in various fields, including healthcare [1], finance [2], and environmental science [3]. In recent years, with the rapid development of machine learning techniques, predictive models trained on time series data have been increasingly deployed across various industries, demonstrating strong application potential. However, due to the typically large number of parameters and the highly nonlinear and complex internal reasoning processes of modern predictive models, their decision-making mechanisms are often difficult for humans to intuitively understand. This not only increases the challenges of model interpretability but also makes it difficult to evaluate whether the features focused on by the model in classification tasks are reasonable. Therefore, improving the transparency and interpretability of models in time series analysis has become an important topic in current research and applications.

To enhance the transparency of models, research on explainable artificial intelligence (XAI) has rapidly advanced [4,5,6]. Currently, researchers have proposed various interpretability methods, including LIME [7] (locally interpretable model-agnostic explanations), IGs [8] (integrated gradients), LRP [9] (layer-wise relevance propagation), and SHAP (SHapley Additive exPlanation) [10]. These methods have been widely applied across different domains. However, these methods are primarily designed for interpreting models trained on image data or text data. Although they can still be used to interpret time series models [11], their effectiveness is limited due to the inherent temporal continuity, sequential nature, and unique frequency domain and time–frequency domain characteristics of time series data.

To address these limitations, XAI research specifically targeting time series data is continuously being conducted [12,13,14]. For example, Sivill et al. [15] proposed the LIMESegment method, which segments time series data into meaningful sub-segments to better capture the temporal and local features of the data. Crabbe et al. [16] proposed the Dynamask method, which generates instance-level feature importance scores by dynamically perturbing masks, ensuring temporal dependency while achieving concise and understandable feature selection, thereby improving interpretability in fields such as healthcare and finance. Queen et al. [17] introduced the TIMEX method, which trains an interpretable surrogate model to mimic the behavior of time series models and addresses the issue of model fidelity by introducing consistency in model behavior. Liu et al. [18] proposed the TIMEX++ algorithm from an information–theoretic perspective. This algorithm is based on the information bottleneck (IB) principle, avoiding trivial solutions and distribution shift problems by optimizing the objective function and using a parameterized network to generate interpretable instances that maintain labels and conform to the data distribution, thereby enhancing the interpretability of time series models.

In recent years, researchers have gradually realized that relying solely on time-domain analysis to interpret time series data has certain limitations [19,20,21]. Therefore, an increasing number of studies have begun to focus on frequency domain and time–frequency domain explanation methods to more comprehensively reveal the intrinsic characteristics and variation patterns of the data. Currently, several methods have been proposed to extend the explanations of traditional interpretability methods to the frequency and time–frequency domains of time series data. For example, Vielhaben et al. [22] proposed the DFT-LRP method, which is based on Fourier transforms. By introducing a virtual inspection layer to convert time series into interpretable representations and applying LRP for attribution analysis, it achieves time–frequency domain explanations for time series data. Chung et al. [23] proposed the SpectralX framework, a time–frequency domain XAI framework capable of providing explanations for black-box time series classification models. It supports multiple perturbation-based analysis methods without requiring modifications to the framework architecture. Additionally, Chung et al. [23] proposed a new perturbation-based XAI method called Feature Importance Approximation (FIA), which improves computational efficiency and enhances class-specific time series interpretability through feature insertion, deletion, and combination techniques.

Although existing research has gradually expanded into the frequency and time–frequency domains, it is still in its early stages of exploration and faces many shortcomings. Taking the SpectralX framework proposed by Chung et al. [23] as an example, although the framework supports nesting multiple perturbation methods and offers strong flexibility, general frameworks often face the issue of “one-size-fits-all”—that is, while pursuing broad applicability, it is difficult to deeply optimize specific methods. The design of SpectralX does not tailor each perturbation strategy specifically and, thus, it cannot ensure that all perturbation methods achieve optimal performance in terms of interpretability.

Based on the above considerations, this paper does not adopt a general framework solution that is highly versatile but lacks specificity. Instead, it chooses to optimize an existing interpretability algorithm by introducing new components and functional modules, making the algorithm more aligned with the analysis requirements of time series in the time–frequency domain, thereby improving the relevance and effectiveness of the explanation results. LIME, as a common model-agnostic interpretability algorithm, has been widely applied in various fields and has demonstrated good performance in practical tasks [24,25,26]. Therefore, this paper builds upon the traditional LIME algorithm and proposes a new interpretability algorithm tailored to the modeling needs of time–frequency features in time series, named time–frequency LIME (TF-LIME). The main contributions of this paper are as follows:

1. Proposal of the time–frequency homogeneous segmentation (TFHS) algorithm. This paper designs a segmentation algorithm for the time–frequency matrix (TFHS), which integrates techniques such as frequency peak detection, spatiotemporal continuity clustering, and dynamic boundary expansion to divide the time–frequency matrix into several homogeneous regions, each corresponding to relatively consistent time–frequency signal characteristics. This method effectively addresses the limitations of traditional LIME in capturing complete homogeneous regions in the time–frequency domain. Compared to methods that treat each time–frequency element as an independent perturbation unit, TFHS significantly reduces the number of perturbations and improves computational efficiency.
2. Proposal of the TF-LIME algorithm integrating multiple techniques. This paper integrates key technologies such as the short-time Fourier transform (STFT) [27], the TFHS segmentation algorithm, LIME, and Inverse STFT (ISTFT) to propose the TF-LIME algorithm, constructing an efficient time–frequency domain explanation framework for time series. This method not only retains the model-agnostic advantages of LIME but also enhances its interpretability and semantic consistency in time–frequency analysis scenarios.
3. Construction of synthetic datasets for algorithm evaluation. This paper designs and constructs two synthetic datasets (Synthetic Dataset 1 and Synthetic Dataset 2) to evaluate the performance of the TFHS segmentation algorithm and the TF-LIME explanation algorithm, respectively. Each dataset is annotated with clear Ground Truth to ensure the objectivity and reproducibility of the evaluation process.
4. Comprehensive Evaluation of Algorithm Performance. The performance of the TFHS segmentation algorithm is quantitatively and qualitatively analyzed on Synthetic Dataset 1. The interpretability of the TF-LIME algorithm is evaluated on Synthetic Dataset 2 and real-world datasets, verifying its effectiveness and robustness in different scenarios.

## 2. Methods

**Figure 1:**
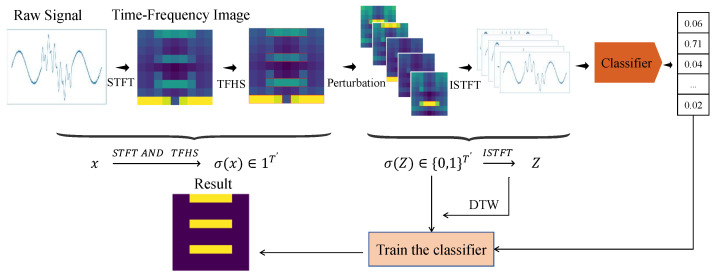
TF-LIME algorithm flowchart.

The TF-LIME algorithm is an extension of the LIME algorithm, designed to perform time–frequency domain analysis on time series data. By utilizing the STFT, this algorithm extracts time–frequency features from time series data, thereby providing a more interpretable analytical approach. The algorithm consists of four key components, namely, interpretable data representation of features, local perturbation, defining the neighborhood, and constructing a surrogate model. The workflow of the TF-LIME algorithm is illustrated in Figure 1.

Without loss of generality, the following assumptions are made: For time series data, a univariate time series sample can be represented as a sequence of *T* observations, denoted as $x=[x_{1},x_{2},\ldots ,x_{T}]$. Given a univariate time series dataset $X\in R^{D\times T}$, where *D* represents the number of samples and *T* represents the number of observations per sample, consider a black-box classifier $f:R^{T}\to R$. For a sample $x\in R^{T}$ whose predicted class is $Y_{x}=f\left(x\right)$, the surrogate model for *x* is denoted as *g*.

### 2.1. Interpretable Data Representation of Features

The TF-LIME algorithm transforms time series data into a comprehensible time–frequency domain by employing the STFT. Through matrix segmentation techniques, it divides the time–frequency matrix into homogeneous regions, thereby obtaining an interpretable data representation σ(x) for the sample *x* under explanation.

#### 2.1.1. The Short-Time Fourier Transform

Time series data often contain rich frequency components, and specific frequency components are closely associated with particular physical characteristics or phenomena [28,29]. Therefore, to achieve an interpretable representation of the sample, *x*, the key lies in uncovering the interpretable features associated with specific categories within the data, which relies on analyzing the evolution of frequencies over time. The STFT, as an effective tool, is capable of precisely capturing the time-varying characteristics of frequencies in time series signals, providing a solid mathematical foundation for this analysis. In the TF-LIME algorithm, the STFT serves as a preliminary step before applying the perturbation method. The equation for STFT is as follows:

(1) $$M\left[l,k\right]=\sum_{n=0}^{N-1}x\left[n+lH\right]\cdot w\left[n\right]\cdot e^{-j2\pi \frac{k}{N}n}$$

Here, M represents the result of the STFT computation, where $M\in C^{L\times K}$. *L* denotes the number of total time segments, and *K* is the total number of frequency bins. Typically, K=N, where *N* is the number of sample points within each analysis window. The variable l∈{0,1,…,L−1} is the index of the time frame, corresponding to successive segments of the input signal shifted by a hop size *H*. Each frame starts at sample lH in the original signal. The variable k∈{0,1,…,K−1} is the index of the discrete frequency bin, each corresponding to a frequency component determined by the discrete Fourier transform (DFT) resolution. x[n+lH] represents the windowed data of the signal at time segment *l*. The exponential term $e^{-j2\pi \frac{k}{N}n}$ serves as the complex sinusoidal basis function in the DFT, associated with the frequency index *k*. The number of time segments *L* is computed as $L=\left\lfloor \frac{T-N}{H}\right\rfloor +1$.

Mathematically, the output of the STFT represents the localized frequency content of the signal over time, providing a joint time–frequency representation of the time series.

#### 2.1.2. Time–Frequency Matrix Segmentation

The time–frequency matrix M of the sample, *x*, under explanation is obtained through the STFT. Each element M[l,k] is a complex-valued coefficient that characterizes the presence of the *k*-th frequency component within the *l*-th time segment. The magnitude |M[l,k]| reflects the local signal energy, while the phase arg(M[l,k]) captures the instantaneous alignment of oscillatory components. Given that each element corresponds to a localized time–frequency region with clear physical semantics [30], the magnitude values of M can be directly used as interpretable features in the LIME framework. This formulation enables LIME to attribute the model’s prediction to specific temporal and spectral components, thereby offering insights into how particular frequency patterns at specific time intervals influence the model’s decision.

**Figure 2:**
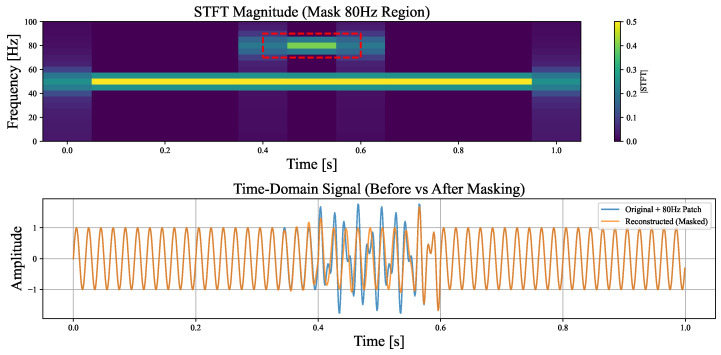
Visualization results of the signal masking experiment.The region enclosed by the red box in the figure corresponds to the masked area.

However, the approach of treating each element in the time–frequency matrix as an independent interpretable unit overlooks the structural continuity and spectral spread of frequency components across the time–frequency domain. In practice, a specific frequency (e.g., 50 Hz) does not manifest in the time–frequency matrix as a single isolated element, but rather as a continuous region extending over time and leaking into adjacent frequency bins due to the finite resolution of the STFT and the effect of spectral leakage [31,32]. To illustrate this, we conduct a simple experiment (Figure 2) where a 50 Hz base signal is corrupted by an 80 Hz interference appearing from 0.4 to 0.6 s. We then attempt to reconstruct or isolate individual frequency components by masking specific regions in the time–frequency matrix. The results show that effective preservation or removal of a particular frequency requires masking a contiguous area that spans not only the full temporal extent but also the surrounding frequency range affected by the component’s spectral leakage. This observation highlights that frequency-based concepts are better represented as structured regions in the time–frequency domain, rather than as individual matrix elements. While perturbing individual time–frequency elements can still produce technically valid explanations, targeting the entire coherent structure formed by a frequency component yields more semantically meaningful results. This is analogous to image interpretation, where it is often more intuitive to interpret a “dog” as a homogeneous region of pixels rather than as a collection of isolated ones. Thus, faithful interpretation or manipulation benefits from operating on such coherent regions.

To achieve refined segmentation of the time–frequency matrix, this paper proposes a segmentation algorithm based on frequency peak detection, spatiotemporal continuity clustering, temporal continuity processing, and dynamic boundary expansion, termed the time–frequency homogeneous segmentation (TFHS) method. This algorithm divides the time–frequency matrix into multiple homogeneous spatiotemporal blocks $B_{n}$, returning a collection of time–frequency blocks *B*, where each block exhibits a certain degree of stability and consistency in both the frequency and time dimensions. The specific steps of the algorithm are detailed below.

(1) Peak detection and clustering: Peaks in the frequency domain of a signal often correspond to specific signal characteristics. Therefore, the first step is to detect these peaks and cluster those representing similar features. Given a time–frequency matrix $M\in R^{L\times K}$, which is the magnitude spectrogram derived from the complex-valued STFT output, significant peaks are identified from the frequency spectrum of each time frame $M_{l,:}$. And a detected peak must satisfy the following conditions:

(2) $$P_{l}=\left\{k|\mathrm{condition}\right\}$$

where

(3) $$\begin{array}{ccc} \; & condition=\\ \; & \; & M_{l,k}\geq \eta \cdot \max\left(M_{l,:}\right)\wedge M_{l,k}\geq \mu_{M}+\beta \cdot \sigma_{M}\\ \; & \; & \wedge M_{l,k}\geq M_{l,k-1}\wedge M_{l,k}\geq M_{l,k+1} \end{array}$$

η is the energy threshold factor. $\mu_{M}$ and $\sigma_{M}$ are the global mean and standard deviation of the time–frequency matrix, respectively. β is the noise suppression coefficient, used to remove low-energy noise. The conditions $M_{l,k}\geq M_{l,k-1}$ and $M_{l,k}\geq M_{l,k+1}$ ensure that the value at frequency index *k* is a local maximum, meaning it is greater than its neighboring frequency points.

Next, the DBSCAN clustering algorithm [33] is applied to the set of peaks $P={⋃}_{l=0}^{L-1}P_{l}$ across all time frames. Prior to clustering, each peak is represented by a frequency–magnitude pair $\left(k,M_{l,k}\right)$, and both dimensions are normalized to ensure equal weighting in distance computation. Clustering is then performed based on these normalized features, aiming to group peaks with similar frequencies and energy levels into the same category. The neighborhood used for clustering is defined as follows:

(4) $$N_{\epsilon }\left(p\right)=\left\{q=\left(k,A\right)|{\left(k_{p}-k_{q}\right)}^{2}+\alpha {\left(A_{p}-A_{q}\right)}^{2}\leq \epsilon^{2}\right\}$$

Here, $k_{p}$ and $k_{q}$ denote the normalized frequency values, while $A_{p}$ and $A_{q}$ denote the normalized magnitude values of peaks *p* and *q*, originally derived from the matrix M. ϵ is the clustering radius, and α is a scaling factor that balances the influence of frequency and magnitude differences. The final clustering result is denoted as $C=\{C_{1},C_{2},\ldots ,C_{n}\}$.

(2) Temporal Continuity Processing: Based on the results of peak clustering, each cluster needs to be processed for temporal continuity. For points within the same cluster, they are sorted by their time indices, and temporal continuity is ensured by requiring that the index gap between adjacent points does not exceed γ:

(5) $$|l_{i}-l_{i-1}|\leq \gamma$$

Here, γ is the maximum allowed time gap. If the interval exceeds this threshold, the cluster is split into multiple temporally continuous subsets. Typically, adjacent points are required to be closely connected.

(3) Dynamic boundary expansion: After obtaining temporally continuous clusters, the left and right boundaries of the frequency band are determined using a bidirectional search method. Specifically, if the time boundaries after temporal continuity processing are $\left[l_{l},l_{r}\right]$ and $\tau =l_{r}-l_{l}+1$, then the left boundary of the frequency band is $k_{\mathrm{low}}$ and the right boundary is $k_{\mathrm{high}}$. The left boundary must satisfy the energy decay constraint:

(6) $$\frac{1}{\tau }\sum_{l=l_{l}}^{l_{r}}M_{l,k_{\mathrm{low}}}\geq \tau_{\mathrm{drop}}\cdot \max_{k\in [k_{low},l_{high}]}\left(\frac{1}{\tau }\sum_{l=l_{l}}^{l_{r}}M_{l,k}\right)$$

and the global energy constraint:

(7) $$\frac{1}{\tau }\sum_{l=l_{l}}^{l_{r}}M_{l,k_{\mathrm{low}}}\geq \tau_{\mathrm{global}}\cdot \mu_{M}$$

Here, $\tau_{\mathrm{drop}}$ is the energy decay constraint, controlling the energy decay ratio at the boundary to avoid inaccuracies in frequency boundaries. $\tau_{\mathrm{global}}$ is the global energy threshold, used to exclude low-energy regions, ensuring the significance and stability of the segmented blocks. The search stops when the frequency value no longer satisfies the above constraints or reaches a local minimum condition, defined as follows:

(8) $$\frac{1}{\tau }\sum_{l=l_{l}}^{l_{r}}M_{l,k}\leq \min\left(\frac{1}{\tau }\sum_{l=l_{l}}^{l_{r}}M_{l,k-1},\frac{1}{\tau }\sum_{l=l_{l}}^{l_{r}}M_{l,k+1}\right),$$

and the current *k* is set as the boundary condition $k_{\mathrm{low}}$. Similarly, the right boundary $k_{\mathrm{high}}$ is determined by satisfying the same constraints.

(4) Time–frequency block generation and optimization: By combining the segmentation results from the frequency and time dimensions, time–frequency blocks $B_{n}=\left(l_{l},l_{r},k_{\mathrm{low}},k_{\mathrm{high}}\right)$ are generated. When time–frequency blocks $B_{n_{1}}$ and $B_{n_{2}}$ have overlapping regions, we reduce the overlapping area by adjusting their boundaries. The boundary condition is defined as follows:

(9) $$\mathrm{overlap}\left(B_{n_{1}},B_{n_{2}}\right)=\frac{|B_{n_{1}}\cap B_{n_{2}}|}{|B_{n_{1}}\cup B_{n_{2}}|}\geq \delta$$

where δ is the overlap threshold. For blocks that meet this condition, their boundaries are adjusted to minimize the intersection area while preserving their main structures. Additionally, time–frequency blocks $B_{n}$ with excessively low average energy are removed, as they are typically induced by noise and may interfere with the construction of matrix blocks.

The above process is based on peak-based time–frequency block partitioning, which primarily divides the time–frequency matrix into blocks where signal energy is concentrated. For the remaining unpartitioned parts of the time–frequency matrix, an equidistant segmentation strategy is adopted to prevent excessively large blocks. By using predefined fixed windows, this method efficiently completes the subsequent segmentation tasks, ensuring the uniformity and stability of the overall segmentation results. Notably, under extreme conditions, the TFHS algorithm defaults to treating each element of the time–frequency matrix as an independent block, ensuring that the algorithm can operate under any circumstances. To further aid understanding, we provide a toy numerical example of the proposed method in Appendix A, which demonstrates the algorithm’s core steps in a simplified setting.

#### 2.1.3. Vector Representation

After obtaining the time–frequency matrix of the time series and completing matrix segmentation, each generated time–frequency block $B_{n}$ represents a homogeneous region, reflecting the intensity and duration of a specific frequency band in the signal. According to the definition provided by Ribeiro et al. [7], interpretable representations should adopt a human-understandable format, even though the model internally may use more complex and less interpretable features. For example, in image classification, an interpretable representation might be a binary vector indicating the “presence” or “absence” of a contiguous pixel block (superpixel), while the classifier might represent the image as a tensor of color channels for each pixel. Here, let $x\in R^{T}$ denote the original representation of the instance being explained, and let $\sigma \left(x\right)\in {\left\{0,1\right\}}^{T^{\prime }}$ represent its interpretable representation. Each value corresponds to a time–frequency block $B_{n}$, where 1 indicates the “presence” of the homogeneous region and 0 indicates its “absence”.

### 2.2. Local Perturbation

After obtaining the interpretable representation of the time series data *x*, generating new samples in its local neighborhood is a challenging problem. The LIME algorithm addresses this by “turning off” specific concepts. To effectively apply this intuition to time series data, it is necessary to define how to “remove” information from the time series. The TF-LIME algorithm achieves this by setting the corresponding elements in σ(x) to zero. Specifically, the TF-LIME algorithm randomly samples non-zero elements from σ(x) and generates new instances around them. At the same time, it ensures that the number of elements sampled each time is uniform, thereby generating a set of perturbed samples, $Z^{\prime }=\left[z_{1}^{\prime },z_{2}^{\prime },\ldots ,z_{u}^{\prime }\right]$.

Next, the perturbed samples need to be restored to their original time-domain representation so that they can be fed back into the model to obtain the model’s prediction probabilities. For a given perturbed sample $z^{\prime }\in {\left\{0,1\right\}}^{T^{\prime }}$ (which contains a subset of the non-zero elements of σ(x)), restoring this sample to its original representation $z\in R^{T}$ requires the use of the ISTFT to convert the time–frequency domain back to the time domain. The ISTFT equation is as follows:

(10) $$z\left[n\right]=\frac{1}{N}\sum_{l=0}^{L-1}\sum_{k=0}^{N-1}M\left[l,k\right]\cdot w\left[n-lH\right]\cdot e^{+j2\pi \frac{k}{N}n}$$

Here, $z\in R^{T}$. The variables are the same as in the STFT. After ISTFT processing, a set of time-domain samples $Z=[z_{1},z_{2},\ldots ,z_{u}]$ will be obtained, where *u* denotes the number of perturbed samples. These restored time-domain signals are then used as inputs to the black-box time series classifier, and the predicted labels $Y_{Z}=f\left(Z\right)$ can be obtained.

### 2.3. Defining the Neighborhood

A key concept in the TF-LIME algorithm is weighting the generated samples as inputs to the interpretable model. In LIME, this weighting is determined by the distance between each new sample and the instance to be explained in their interpretable representations. The TF-LIME algorithm follows the same idea. For time series data, it is necessary to consider how to measure the distance between two time series to accurately reflect the local neighborhood around *x*. The dynamic time warping (DTW) algorithm provides a solution for this.

DTW is a nonlinear sequence alignment algorithm used to measure the similarity between two time series [34]. Unlike traditional Euclidean distance, DTW allows for elastic deformation along the time axis, enabling optimal matching even when the sequences exhibit nonlinear temporal variations. By using the DTW, the similarity between the sample *x* to be explained and each sample *z* in the time series set *Z* is calculated as DTW(x,z). To eliminate differences in the magnitude and distribution of DTW distances across samples, normalization is applied to obtain ${\mathrm{DTW}}_{z-\mathrm{norm}}\left(Z\right)$. Subsequently, ${\mathrm{DTW}}_{z-\mathrm{norm}}\left(Z\right)$ is input into an exponential kernel function with scale parameter ρ, and the weight is calculated using the formula:

(11) $$\pi_{x}\left(z\right)=\exp\left(-\frac{{\mathrm{DTW}}_{z-\mathrm{norm}}{\left(Z\right)}^{2}}{\rho^{2}}\right)$$

This weight reflects the similarity between samples *z* and *x*, allowing the local interpretable model to assign higher weights to samples closer to *x*. This ensures that the model better approximates the behavior of the black-box model in the vicinity of *x*.

### 2.4. Constructing a Surrogate Model

The goal of the TF-LIME algorithm is to construct a surrogate model *g* in the local neighborhood of a given time series instance *x* to be explained, along with the black-box classifier $f:R^{T}\to R$ and the predicted label $Y_{x}=f\left(x\right)$. This surrogate model *g* generates explanations in the interpretable domain σ(x).

By default, the TF-LIME algorithm uses linear ridge regression as the surrogate model *g*, and the feature weight vector $w=[w_{1};\ldots ;w_{T^{\prime }}]$ is interpreted as the importance of each homogeneous region.

## 3. Experimental Results

This section aims to experimentally validate the performance of the TFHS algorithm in time–frequency segmentation tasks and the interpretability effectiveness of the TF-LIME algorithm in time series classification tasks. The experimental design includes the following main parts: First, the datasets used are introduced, including synthetic data and real-world data, along with a brief description of the models trained on these datasets. Next, the comparative methods, parameter configurations, and evaluation metrics used in the experiments are detailed. Subsequently, based on synthetic datasets, the segmentation capability of the TFHS algorithm is verified. Finally, the effectiveness of the TF-LIME algorithm in explaining the decision-making of classification models is systematically evaluated on both synthetic and real-world datasets.

### 3.1. Dataset and Model Introduction

To validate the effectiveness of the TFHS algorithm and the TF-LIME algorithm, both synthetic and real-world datasets are used in the experiments. Below, we provide a detailed introduction to the roles of each dataset in the experiments, their key characteristic parameters, and the model architectures designed for different tasks.

#### 3.1.1. Synthetic Dataset 1

Synthetic Dataset 1 is used to evaluate the effectiveness of the time–frequency matrix segmentation algorithm and is not used for model training. The signals in this dataset are composed of the superposition of *K* simple harmonic sine waves, and their mathematical model can be described as follows:

(12) $$f\left(t\right)=\underset{\mathrm{deterministic}\;\mathrm{component}}{\underset{︸}{\sum_{k=1}^{K}A_{k}\sin\left(2\pi f_{k}t+\Phi_{k}\left(t\right)\right)W_{k}\left(t\right)}}+\underset{\mathrm{random}\;\mathrm{noise}}{\underset{︸}{\sigma \eta (t)}}$$

where $W_{k}\left(t\right)$ is the time window function, defined as follows:

(13) $$W_{k}\left(t\right)=u\left(t-\tau_{k}^{\mathrm{start}}\right)-u\left(t-\tau_{k}^{\mathrm{end}}\right)$$

and u(t) is the unit step function:

(14) $$u\left(t\right)=\left\{\begin{array}{cc} 1, & t\geq 0,\\ 0, & t<0. \end{array}\right.$$

In the above equations, t∈R represents the continuous time variable, *K* is the total number of signal components, $A_{k}$ is the amplitude of the *k*-th signal component, $f_{k}$ is its fundamental frequency, and $\Phi_{k}\left(t\right)$ is the time-varying phase function (by default, $\Phi_{k}\left(t\right)=\phi_{k}$ is a constant phase). $W_{k}\left(t\right)$ is used to define the activation time interval of the signal component, σ is the noise intensity coefficient, and η(t) represents Gaussian white noise following a standard normal distribution N(0,1). Additionally, $\tau_{k}^{\mathrm{start}}$ and $\tau_{k}^{\mathrm{end}}$ represent the start and end times of the *k*-th signal component, respectively.

Specifically, the dataset contains 200 single-frequency signal samples and 800 multi-frequency signal samples, totaling 1000 samples. Each sample consists of 1000 data points. The total number of signal components *K* varies between 1 and 5 to simulate signals of different complexities. The fundamental frequency $f_{k}$ ranges from 0 to 50 Hz, and $A_{k}$ varies between 1 and 5 to ensure diversity in signal strength. The phase $\phi_{k}$ follows a uniform distribution U(0,2π) to ensure randomization of the signal component phases. The time window parameters $\tau_{k}^{\mathrm{start}}$ and $\tau_{k}^{\mathrm{end}}$ follow a uniform distribution to control the activation duration of the signals, ensuring that different signal components are distributed randomly along the time axis. The noise intensity coefficient is set to σ=0.01 and σ=0.7 to adjust the noise level of the signals, simulating signal interference in real-world environments. The continuous signals are sampled at 200 Hz. To quantify the performance of the time–frequency matrix segmentation algorithm, the changes in the time–frequency matrix corresponding to each new frequency component added to the original data are recorded during the construction of the dataset. This information serves as the ground truth, which includes the main energy of the original data and is used for subsequent experimental validation and algorithm evaluation.

#### 3.1.2. Synthetic Dataset 2

Synthetic Dataset 2 is used to quantify the effectiveness of the TF-LIME algorithm in explaining time–frequency domain characteristics. The signals in this dataset are composed of the superposition of *K* simple harmonic sine waves, and their mathematical model can be represented by Equation (12). This dataset is based on a time–frequency joint detection task, which requires the model to identify the frequency combinations contained in different time windows across various signals. All possible frequency combinations are derived from a predefined frequency set $k^{*}=\left\{k_{1},k_{2},k_{3}\right\}$, with specific frequency combinations, including the empty set {}, single-frequency combinations $\left\{k_{1}\right\}$, binary combinations $\left\{k_{1},k_{2}\right\}$, and the full-frequency combination $\left\{k_{1},k_{2},k_{3}\right\}$, among 8 types in total. Each frequency combination may or may not appear within the fixed time windows $T^{*}=\left\{T_{1},T_{2}\right\}$. The frequency combinations are arranged in a permutation manner along the time dimension rather than in a combinatorial manner, resulting in a total of 64 different labels in the dataset. Given the simplicity of the task, we assume that the true explanation should only attribute positive correlations to the time–frequency regions closely associated with the $\left(k^{*},T^{*}\right)$ subsets corresponding to the labels. Based on this assumption, the ground truth for the explanations of Synthetic Dataset 2 is generated. By comparing the explanations generated by TF-LIME with these predefined ground truths, the algorithm’s fidelity in capturing the time–frequency characteristics of the signals can be precisely quantified.

Specifically, in the experiment, we selected $k^{*}=\left\{5,20,40\right\}$ as the frequency set, where these three frequencies represent the main components that may appear in the signal. $T^{*}=\left\{\left(0,2.5\;s\right),\left(2.5\;s,5\;s\right)\right\}$ is used as the time segmentation, with these two time intervals representing the two regions where composite signals may appear. The sampling frequency is 200 Hz, the sampling duration is 5 s, and the sample length is N=1000. Other parameters remain consistent with those in Synthetic Dataset 1.

For model training, a simple multilayer perceptron (MLP) model [35] with two hidden layers and ReLU activation functions was employed [36]. During the training phase, the model was first trained on $2\times 10^{4}$ samples for the baseline task (σ=0.01). Subsequently, noise was introduced, and the model was retrained under the condition of σ=0.7. Experimental results show that the MLP model achieved excellent performance on 1000 test samples: under low-noise conditions (σ=0.01), the model achieved an accuracy of 94.7%, while under higher-noise conditions (σ=0.7), the model achieved an accuracy of 93.4%.

#### 3.1.3. MIT-BIH Dataset

The MIT-BIH Arrhythmia Dataset [37] will be used to demonstrate the performance of the TF-LIME algorithm on real-world data. The MIT-BIH dataset contains electrocardiogram (ECG) recordings from 47 subjects, with a sampling rate of 360 Hz. Each heartbeat is annotated by at least two cardiologists. Before training the model, the ECG data undergoes preprocessing. The preprocessing follows the work of Kachuee et al. [38], which isolates the ECG lead II data, resamples it at 125 Hz, and segments and pads it into fixed-length individual heartbeats of 1500 ms.

Additionally, this study adopts the 1D-CNN heartbeat classification model proposed by Kachuee et al. [38]. The model takes the preprocessed heartbeat signals as input, and its core architecture consists of 5 residual blocks [39]. Each residual block contains two convolutional layers (with a kernel size of 5 and 32 filters), two ReLU activation functions, a residual skip connection, and a max-pooling layer (with a kernel size of 5 and a stride of 2). After feature extraction, the network further includes two fully connected layers (each with 32 neurons), and finally outputs the probability distribution of heartbeat categories through a Softmax layer. Experimental results show that this model achieves an accuracy of 95.3% on the heartbeat classification task.

### 3.2. Baselines

We compare the TF-LIME algorithm with other commonly used local attribution methods, including sensitivity [40], integrated gradients (IGs) [8], and ε-rule-based layer-wise relevance propagation (LRP) [9]. Since these methods are typically only applicable to time-domain analysis of time series, we employ the “virtual hidden layer” technique [22] to extend interpretability to the time–frequency domain. Specifically, an inverse (ST)DFT layer is appended before the input layer, enabling the new model to directly propagate importance to the time–frequency domain; here, the signal is split into real and imaginary parts. All attribution methods are implemented using the code provided in the Zennit software package [41].

Additionally, we compare the TF-LIME algorithm with perturbation-based interpretability methods, including LIME [7] and feature importance approximation (FIA) [23]. For this purpose, we use the Spectral eXplanation (SpectralX) framework proposed by Chung et al. [23]. This is an explainable artificial intelligence (XAI) framework designed to provide time–frequency domain explanations for black-box time series classifiers. The framework is highly adaptable, allowing users to easily “plug in” different perturbation-based XAI methods to evaluate their impact on explanation quality without modifying the framework’s architecture.

### 3.3. Parameter Settings

To facilitate the reproduction of the experimental process by readers, Table 1 summarizes the main parameter settings used in this paper, which serve as the default configurations throughout our experiments. For different datasets, modifications to certain parameters are briefly described in the corresponding sections; if not specified, the default settings are applied. Additionally, to assist readers in adapting the algorithm to different datasets, Appendix B provides further discussion on parameter usage and recommended configurations.

In particular, the Hann window is consistently applied in both STFT and FFT computations to reduce spectral leakage, following standard signal processing practices.

### 3.4. Metrics

In the experiments, we employ different evaluation metrics to quantify the actual performance of the algorithms. Specifically, these evaluation metrics can be divided into two categories: one for quantifying the performance of the TFHS algorithm and the other for assessing the effectiveness of the TF-LIME algorithm.

Metrics for evaluating TFHS: To quantitatively evaluate the alignment between the segmentation results of the TFHS algorithm and the ground truth, the following three metrics are adopted:

- Intersection over union (IoU) [42]: Measures the overlap between the predicted segmentation region and the ground truth segmentation region, defined as follows:
  (15) $$IoU=\frac{\left|P\cap G\right|}{\left|P\cup G\right|}$$
  where *P* represents the predicted segmentation region, *G* represents the ground truth segmentation region. For multi-class segmentation problems, the mean IoU (mIoU) is calculated by averaging the IoU values across all classes:
  (16) $$\mathrm{mIoU}=\frac{1}{N}\sum_{X}IoU_{X}$$
  where *N* is the number of classes, and $IoU_{X}$ is the IoU value for class *X*.
- False positive rate (FPR): Measures the proportion of incorrectly extracted regions in the algorithm’s output, i.e., regions that are not part of the ground truth but are mistakenly identified by the algorithm:
  (17) $$FPR=\frac{\left|P\setminus G\right|}{\left|P\right|}$$
  where P∖G represents the regions in the predicted segmentation that are not marked as signals in the ground truth. A higher FPR indicates more false detections, leading to increased noise impact.
- Energy retention ratio (ERR): Calculates the proportion of energy in the overlapping region between the predicted segmentation and the ground truth relative to the total energy in the ground truth. This metric evaluates whether the segmentation accurately captures the signal energy:
  (18) $$ERR=\frac{\sum_{(x,y)\in P\cap G}E\left(x,y\right)}{\sum_{(x,y)\in G}E\left(x,y\right)}$$
  where E(x,y) is the signal energy value at position (x,y) in the matrix. If ERR=1, it indicates that the segmentation region fully covers the high-energy regions of the ground truth; if ERR<1, it indicates that some signal energy is not captured.

Metrics for evaluating the TF-LIME algorithm: To quantitatively evaluate the alignment between the explanations generated by the model and the ground truth, we use the area under precision (AUP) and area under recall (AUR) curves to evaluate the quality of explanations [16]. We also employ the explanation area under the precision–recall curve (AUPRC), which combines the results of the two aforementioned metrics [17,18]. For all metrics, higher values indicate better performance. The calculation process of AUP and AUR is as follows:

Let *Q* be a matrix with elements in ${\left\{0,1\right\}}^{T\times d_{X}}$, indicating the ground truth significance of the input data $x\in R^{T\times d_{X}}$, i.e., the ground truth of the time–frequency matrix. By definition, when the feature $x_{t,i}$ is significant, $Q_{t,i}=1$; otherwise, $Q_{t,i}=0$.

Let *M* be a mask matrix with elements in ${\left\{0,1\right\}}^{T\times d_{X}}$, generated by a significance method. Let τ∈(0,1) be a detection threshold used to determine whether $M_{t,i}$ indicates that the feature $x_{t,i}$ is significant. Thus, we can convert the mask into an estimated matrix ${\hat{Q}}_{t,i}\left(\tau \right)$:

(19) $${\hat{Q}}_{t,i}\left(\tau \right)=\left\{\begin{array}{cc} 1, & \mathrm{if}\;M_{t,i}\geq \tau \\ 0, & \mathrm{otherwise} \end{array}\right.$$

Define the set of indices for truly significant features *A* and the set of indices selected by the significance method $\hat{A}\left(\tau \right)$:

(20) $$A=\{\left(t,i\right)\in \left[1:T\right]\times \left[1:d_{X}\right]|Q_{t,i}=1\}$$

(21) $$\hat{A}\left(\tau \right)=\left\{\left(t,i\right)\in \left[1:T\right]\times \left[1:d_{X}\right]|{\hat{Q}}_{t,i}\left(\tau \right)=1\right\}$$

Next, we define the precision and recall curves, mapping each threshold to the corresponding precision and recall:

(22) $$P:\left(0,1\right)\to \left[0,1\right],\;\tau \mapsto \frac{|A\cap \hat{A}\left(\tau \right)|}{|\hat{A}\left(\tau \right)|}$$

(23) $$R:\left(0,1\right)\to \left[0,1\right],\;\tau \mapsto \frac{|A\cap \hat{A}\left(\tau \right)|}{\left|A\right|}$$

The area under the precision curve (AUP) and the area under the recall curve (AUR) are the areas under these curves, respectively:

(24) $$AUP=\int_{0}^{1}P\left(\tau \right)d\tau$$

(25) $$AUR=\int_{0}^{1}R\left(\tau \right)d\tau$$

### 3.5. Evaluation of the TFHS Algorithm

This section quantitatively evaluates the segmentation performance of the TFHS algorithm on the 1 by using the metrics mentioned in Section 3.4. Additionally, qualitative evaluation is conducted using visualization techniques to further demonstrate the algorithm’s capabilities.

#### 3.5.1. Quantitative Evaluation

In Synthetic Dataset 1, the ground truth for the explanations of each sample is recorded. Therefore, we use the evaluation metrics introduced in Section 3.4 to quantitatively assess the effectiveness of the TFHS algorithm.

The quantitative evaluation results of the TFHS algorithm are shown in Table 2. Through data analysis, it can be observed that the TFHS algorithm demonstrates excellent time–frequency feature extraction capabilities under low-noise conditions (σ=0.01). For single-frequency samples, the IoU reaches 93%, and the ERR exceeds 94%, indicating its ability to accurately capture the time–frequency energy distribution of the signals. When the noise level increases to σ=0.7, the IoU and ERR for all samples decrease by 4–6 percentage points and 3–5 percentage points, respectively, while the FPR increases by 1–3 percentage points. This is primarily due to two types of errors caused by noise interference: on one hand, high-frequency noise in the time–frequency plot overlaps with signal components, increasing the probability of false alarms. On the other hand, weak energy signal regions are drowned by noise, leading to higher missed detection rates. Notably, the IoU for multi-frequency samples remains at a relatively high level under high-noise conditions, confirming the algorithm’s substantial noise resistance.

The performance difference between single-frequency and multi-frequency samples reveals the fundamental challenge of time–frequency segmentation—the negative correlation between signal complexity and segmentation accuracy. Single-frequency signals, due to their smooth time–frequency trajectories and energy concentration, achieve an IoU of up to 93% using rectangular windows. In contrast, multi-frequency signals exhibit local energy diffusion caused by cross-modulation of different frequency components, resulting in non-stationary time–frequency distributions. This makes it difficult for fixed-shape segmentation windows to precisely match the true energy contours. Further analysis shows that approximately 68% of segmentation errors originate from signal mutation regions (e.g., frequency switching points or amplitude jump points). The spectral energy diffusion (Gibbs phenomenon [43]) in these regions causes energy leakage at the edges of time–frequency blocks, and the traditional rectangular windows are insufficiently adaptive to such nonlinear features, leading to increased missed detection rates in edge regions.

Despite the impact of edge effects, the ERR metric indicates that the energy loss in uncaptured regions remains consistently low, demonstrating that the TFHS algorithm can effectively preserve the main characteristics of the signals. From a system-level perspective, since time–frequency analysis tasks focus more on locating dominant energy regions (rather than strictly precise boundary delineation), the algorithm’s precision-robustness balance already meets the requirements for subsequent interpretability analysis in TF-LIME. In the future, introducing adaptive morphological windows and edge compensation strategies could further mitigate the impact of the Gibbs phenomenon on segmentation accuracy.

#### 3.5.2. Visualization

**Figure 3:**
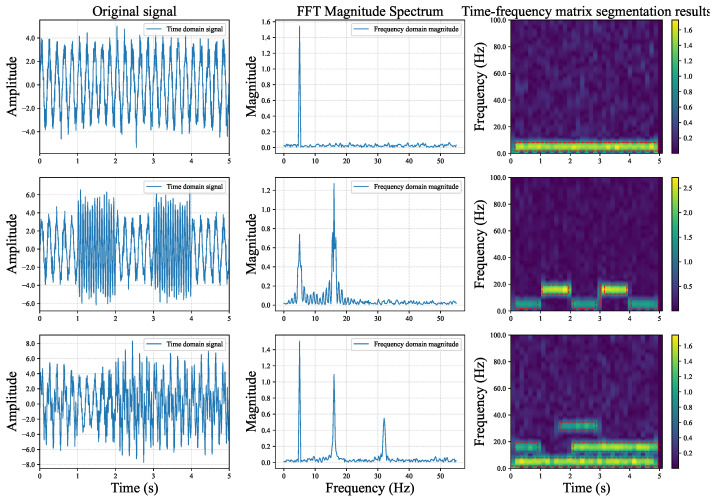
Segmentation results of TFHS algorithm on 1.

Synthetic Dataset 1 helps us quantify the effectiveness of the TFHS algorithm. To better demonstrate the performance of the TFHS algorithm, we selected a subset of data from Synthetic Dataset 1 for visualization. The results are shown in Figure 3. It can be observed that the TFHS segmentation algorithm not only captures the changes of different signals in the time domain, but also captures the different frequency components contained in the signal in the frequency domain, and performs well.

### 3.6. Evaluation of the TF-LIME Algorithm on Synthetic Datasets

This section evaluates the explanation effectiveness of the TF-LIME algorithm on time–frequency detection models trained on both baseline and noise tasks. The specific tasks and trained models have been detailed in Section 3.1.2 under Synthetic Dataset 2. In the experiments, the parameter ϵ is set to 0.6.

#### 3.6.1. Quantitative Evaluation

Table 3 and Table 4 present the performance metrics of the TF-LIME algorithm under baseline and noisy environments, respectively. The experimental results demonstrate that TF-LIME exhibits significant advantages in providing time–frequency domain explanations for time series models, outperforming traditional gradient-based attribution methods and perturbation-based approaches. In the following, we will analyze the experimental results and further discuss the superiority of TF-LIME in this scenario.

First, it can be observed from the experimental data that the sensitivity method consistently underperforms compared to other methods. This is likely because sensitivity relies solely on gradient calculations for attribution scores, emphasizing local effects while neglecting the overall characteristics of the signal [44,45]. Meanwhile, the experimental results for LRP and IG are highly similar, which may be attributed to the relatively simple model structure used in the experiments. The model consists of only two hidden layers with ReLU as the activation function, resulting in low nonlinearity and an overall linear approximation, thus causing LRP and IG to produce close attribution scores.

In contrast, perturbation-based methods such as LIME and FIA exhibit different performance in this task. Experimental results show that LIME can train an effective surrogate model to fit local features without requiring a large number of perturbed samples, which may also be attributed to the overall approximate linearity of the model. However, LIME’s strategy of treating each element in the time–frequency matrix as an independent interpretable unit has two limitations. On one hand, it ignores the temporal continuity of features, only perturbing signals within each time window. On the other hand, in the frequency domain, a single matrix element cannot fully capture the complete energy information of the features, which affects the quality of the explanation results. Additionally, another perturbation-based method, FIA (frequency-based interpretability attack), which is specifically designed for frequency domain analysis, has limited applicability in this task. We found that the RBP [15] (Realistic Background Perturbation) technique introduced in FIA is based on the assumption that there exists a stable “background” frequency in the data. However, for time–frequency signals, this assumption does not always hold. A background as defined by RBP can only exist when repeated frequencies are present in two time windows, and in our dataset, such cases account for only 12.5% of the total samples, which is a relatively low proportion. As a result, RBP fails to achieve the expected performance in this task. Experimental results also show that FIA-Deletion, which does not use RBP, outperforms the two RBP-based FIA variants across all metrics.

To address these limitations, TF-LIME divides the time–frequency matrix into homogeneous regions, grouping similar areas into the same blocks. This approach aligns the explanations more closely with the intrinsic characteristics of the signal, resulting in significant advantages across all metrics. In comparative experiments between baseline and noisy environments, TF-LIME maintains its performance superiority even under noisy conditions, further validating its adaptability and stability in complex environments.

#### 3.6.2. Visualization

**Figure 4:**
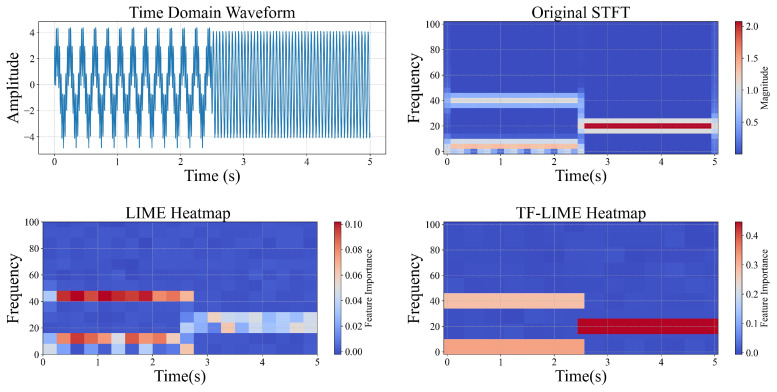
Synthetic Signal 2 heatmap visualization.

Figure 4 presents the visualization of the interpretative results of the TF-LIME algorithm on a test sample from Synthetic Dataset 2. The true label of this sample is {(5,40), (20)}, indicating the presence of frequency components at 5 Hz, 20 Hz, and 40 Hz during different time intervals. For comparison, the explanation heatmap generated by the traditional LIME algorithm for the same sample is also provided, to analyze the differences in temporal feature interpretability between the two methods.

From the figure, it can be observed that while the traditional LIME method partially covers the target frequency bands, the activated regions are scattered and include many redundant explanations unrelated to the actual targets. In contrast, TF-LIME aligns more precisely with the target structures in both frequency and time dimensions, and its heatmap exhibits stronger sparsity and readability, effectively highlighting key time–frequency regions. Overall, the interpretative results of TF-LIME show high consistency with the original STFT energy distribution, validating its superior local interpretability in time series classification tasks.

### 3.7. Evaluation of the TF-LIME Algorithm on Real-World Datasets

This section evaluates the effectiveness of the TF-LIME algorithm on a real-world dataset, using the MIT-BIH Arrhythmia Database as the experimental benchmark. The specific classification task and the model used for interpretation have been detailed in Section 3.1 and are therefore omitted here. Since the time–frequency signal strength of the MIT-BIH data after preprocessing is significantly weaker than that of the synthetic dataset, adjustments were made to the DBSCAN clustering parameters to ensure stable and discriminative performance. Specifically, the clustering radius was set to ϵ=0.32 and η=0.25. All other parameters remain consistent with the default settings described in Section 3.3.

#### 3.7.1. Quantitative Evaluation

Table 5 presents the attribution interpretation performance of different explainability methods on the MIT-BIH dataset. The experimental results demonstrate that the TF-LIME algorithm achieves the best performance in both AUPRC and AUR metrics, while its AUP metric is also close to the optimal result, showcasing strong attribution accuracy and stability overall.

Furthermore, by comparing the experimental results based on simple models in Section 3.6, it can be observed that as the model complexity increases and the degree of nonlinearity intensifies, the explanatory power of the traditional LIME algorithm significantly declines. We believe this is primarily due to LIME’s attribution mechanism relying on treating individual input dimensions (such as feature points or pixels) as the smallest interpretable units, resulting in high-dimensional interpretable representations σ(x). Even when attempting to partition the time–frequency matrix using fixed windows, it is often difficult to effectively cover key homogeneous regions, limiting the expressiveness of the attribution results. In contrast, TF-LIME introduces structured interpretable units in the time–frequency domain, effectively compressing the dimensionality of the explanation space and improving attribution quality and semantic consistency.

At the same time, high-dimensional explanation spaces also impose higher requirements on the number of perturbation samples. Traditional LIME typically requires generating a large number of perturbation samples to achieve stable attribution results in high-complexity models, which significantly increases the computational burden in practical applications. In this experiment, LIME used 500 perturbation samples for attribution. In comparison, TF-LIME can achieve more discriminative and consistent attribution outputs with a smaller sample size, striking a good balance between efficiency and effectiveness, demonstrating its advantages in high-complexity model environments.

#### 3.7.2. Visualization

**Figure 5:**
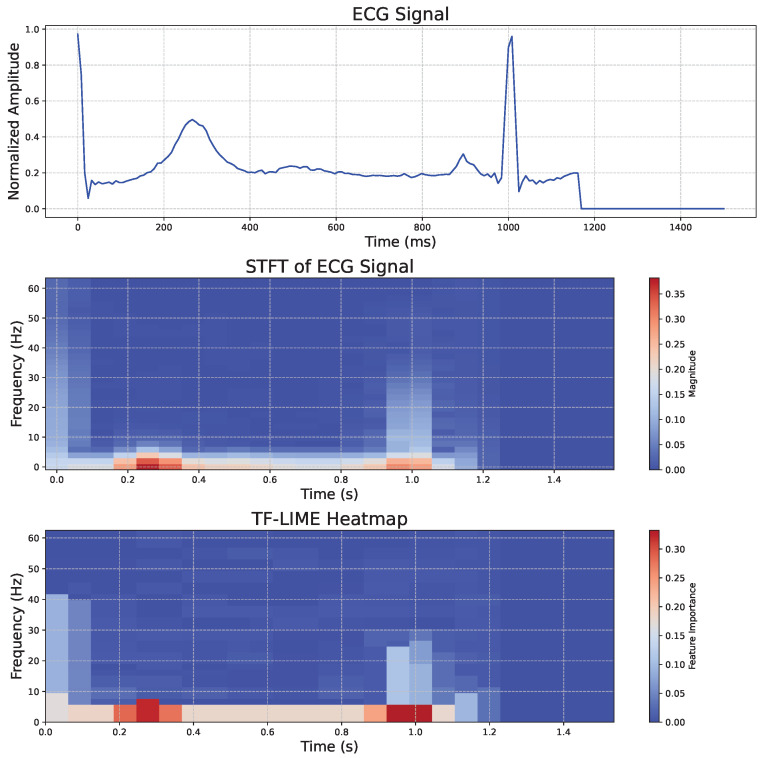
ECG signal heatmap visualization.

To help readers better understand the ECG waveform obtained after preprocessing in Section 3.1.3 and to demonstrate the interpretability of TF-LIME on real-world data, Figure 5 presents a representative ECG signal analysis, including the original waveform (top), the corresponding STFT spectrogram (middle), and the feature importance heatmap generated by the TF-LIME algorithm (bottom).

As shown in Figure 5, the important regions identified by TF-LIME are highly concentrated in frequency bands corresponding to abrupt changes in signal amplitude. The heatmap indicates that the model focuses primarily on regions associated with key ECG features, such as the QRS complex, as well as the T and P waves, when making predictions.

## 4. Conclusions

This paper proposes a model-agnostic interpretability method for time-series data, namely the TF-LIME algorithm. This method breaks through the limitations of traditional time-domain explanations and innovatively extends the interpretative perspective to the time–frequency domain, providing more comprehensive explanatory capabilities for time series models.

The core of the TF-LIME algorithm lies in its use of the STFT and its inverse transform (ISTFT) to achieve bidirectional conversion between the time domain and the time–frequency domain. It also introduces an innovative time–frequency matrix segmentation technique (TFHS algorithm) to identify homogeneous regions within the time–frequency matrix. Based on this, the algorithm can accurately quantify the significance of each homogeneous region’s contribution to the model’s predictions through perturbation sampling and linear fitting methods.

Through systematic experimental validation on synthetic datasets and the MIT-BIH dataset, the TF-LIME algorithm demonstrates good interpretative performance in the time–frequency domain. Compared to the traditional LIME algorithm, TF-LIME not only retains time-domain interpretability but also significantly enhances the comprehensiveness and reliability of model explanations by incorporating time–frequency domain features. The experimental results show that this method provides a new technical approach for interpreting time series models, with important theoretical significance and practical value.

Future research will focus on the interpretability of multidimensional time series models, emphasizing the following directions:

- Multidimensional dynamic correlation modeling: There is a need to develop interpretative tools that can simultaneously capture temporal dimensions and interactions between variables, breaking through the limitations of traditional univariate analysis. This will provide new possibilities for a deeper understanding of complex time series data.
- Construction of a general interpretative framework: The goal is to establish a model-agnostic standardized interpretative interface that is compatible with mainstream time series models such as RNNs and Transformers. This will significantly improve the applicability and transferability of the method.
- Validation in multidisciplinary applications: Research will test the proposed method in typical scenarios such as medical monitoring (e.g., multi-parameter physiological signals) and industrial sensing (e.g., multi-sensor data from equipment) to verify its reliability and robustness.

## Figures and Tables

**Table 1:** Experimental parameter settings.

Parameter	Value
Energy threshold factor η	0.5
Noise suppression coefficient β	1.5
DBSCAN clustering radius ϵ	0.5
Scaling factor α	1
Maximum time gap γ	1
Energy decay constraint $\tau_{\mathrm{drop}}$	0.6
Global energy threshold $\tau_{\mathrm{global}}$	1.2
Overlap threshold δ	0.3
Segmentation window window	2
Number of generated perturbation samples *u*	500

**Table 2:** The quantitative evaluation results of the TFHS algorithm (mean).

Sample Type	IoU (%)	FPR (%)	ERR (%)
Single-frequency (σ=0.01)	93.1	2.3	94.7
Single-frequency (σ=0.7)	85.5	3.3	92.5
Multi-frequency (σ=0.01)	83.1	6.2	89.2
Multi-frequency (σ=0.7)	79.3	8.6	84.3

**Table 3:** The attribution explanation performance of different interpretability methods on Synthetic Dataset 2 (Baseline, σ=0.01).

Method	AUPRC	AUP	AUR
Sensitivity	0.34	0.43	0.32
IG	0.56	0.62	0.44
LRP	0.57	0.61	0.45
FIA-Insertion	0.42	0.41	0.35
FIA-Deletion	0.58	0.57	0.37
FIA-Combined	0.52	0.56	0.36
LIME	0.64	0.61	0.54
TF-LIME	0.83	0.78	0.64

**Table 4:** The attribution explanation performance of different interpretability methods on Synthetic Dataset 2 (Noise, σ=0.7).

Method	AUPRC	AUP	AUR
Sensitivity	0.30	0.38	0.28
IG	0.52	0.55	0.41
LRP	0.50	0.54	0.40
FIA-Insertion	0.41	0.38	0.33
FIA-Deletion	0.53	0.51	0.34
FIA-Combined	0.49	0.49	0.34
LIME	0.59	0.52	0.47
TF-LIME	0.75	0.72	0.60

**Table 5:** The attribution explanation performance of different interpretability methods on the MIT-BIH dataset.

Method	AUPRC	AUP	AUR
IG	0.42	0.59	0.39
LRP	0.47	0.61	0.41
FIA-Insertion	0.31	0.42	0.29
FIA-Deletion	0.43	0.57	0.40
FIA-Combined	0.39	0.49	0.37
LIME	0.38	0.49	0.36
TF-LIME	0.49	0.60	0.43

## Data Availability

Requests for access to the data supporting the results of this study be directed to the corresponding author via email (itswzz8@tju.edu.cn).

## Appendix A. Toy Numerical Example of the Proposed Method

To illustrate the full process of the proposed method in a concrete and intuitive way, we provide a toy numerical example using a simplified time–frequency matrix. This example is not used for model training or evaluation, but purely for explanatory purposes. To offer a more detailed explanation, we further decompose the original four main steps into finer-grained sub-steps.

### Appendix A.1. Time–Frequency Matrix Construction

We begin by considering a toy example of a time–frequency magnitude matrix $M\in R^{9\times 9}$, representing 9 frequency bins across 9 time frames. The matrix is defined as follows:

$$M=\left[\begin{array}{ccccccccc} 0.10 & 0.10 & 0.10 & 0.01 & 0.01 & 0.01 & 0.10 & 0.10 & 0.10\\ 0.30 & 0.32 & 0.33 & 0.11 & 0.10 & 0.12 & 0.32 & 0.31 & 0.33\\ 0.80 & 0.82 & 0.81 & 0.30 & 0.32 & 0.31 & 0.83 & 0.81 & 0.82\\ 0.30 & 0.32 & 0.33 & 0.11 & 0.10 & 0.12 & 0.32 & 0.31 & 0.33\\ 0.10 & 0.10 & 0.10 & 0.01 & 0.01 & 0.01 & 0.10 & 0.10 & 0.10\\ 0.01 & 0.01 & 0.01 & 0.22 & 0.21 & 0.20 & 0.01 & 0.01 & 0.01\\ 0.01 & 0.01 & 0.01 & 0.72 & 0.75 & 0.70 & 0.01 & 0.01 & 0.01\\ 0.01 & 0.01 & 0.01 & 0.22 & 0.21 & 0.20 & 0.01 & 0.01 & 0.01\\ 0.01 & 0.01 & 0.01 & 0.01 & 0.01 & 0.01 & 0.01 & 0.01 & 0.01 \end{array}\right]$$

Each column $M_{:,l}$ represents the frequency spectrum at the *l*-th time frame. The matrix is arranged such that the frequency increases from top to bottom, while time progresses from left to right. Note that the notation $M_{:,l}$ used here is equivalent in meaning to $M_{l,:}$ as used in the original formulation; the change in notation is made purely for ease of understanding within the context of the time–frequency matrix layout.

This matrix incorporates typical characteristics often encountered in time–frequency representations, such as spectral leakage and temporal smearing. It serves as an idealized example constructed solely for the purpose of illustrating the TFHS algorithm. The parameter settings used in the description are designed exclusively for explanatory purposes and do not reflect any specific experimental configuration. For actual implementations, please refer to the detailed parameter guidelines provided in Appendix B. The annotated values indicate the presence of local spectral peaks at specific time frames. The use of black and red highlights is intended solely for visual emphasis and does not convey any semantic distinction.

### Appendix A.2. Peak Detection

The peak detection process is carried out in two sequential stages, detailed as follows.

(1) Local Peak Identification

For each time frame *l*, we extract the corresponding column vector $M_{:,l}$ and detect local maxima along the frequency axis. A point $M_{k,l}$ is considered a local peak if it satisfies:

$$M_{k,l}>M_{k-1,l}\;\mathrm{and}\;M_{k,l}>M_{k+1,l}$$

All such local peaks are collected into an initial candidate set $P_{l}$ for each frame.

To further eliminate low-amplitude fluctuations that may be caused by noise or weak background signals, each candidate peak is subjected to an additional filtering criterion based on its relative amplitude within the current frame. Specifically, a local peak $M_{k,l}$ is retained only if it satisfies:

$$M_{k,l}\geq \eta \cdot \max\left(M_{:,l}\right)$$

This step ensures that only prominent peaks, with sufficient energy compared to the dominant frequency component in the frame, are included in the final set. In our example, after applying the above criteria, the retained local peaks correspond to the following time–frequency matrix indices:

{(2,0),(2,1),(2,2),(2,3),(2,4),(2,5),(2,6),(2,7),(2,8),(6,3),(6,4),(6,5)}

For instance, in the first column, the value 0.80 satisfies the condition 0.80>0.30 and 0.80>0.30, indicating it is a valid local peak(corresponding to the index (2,0)). All other values in this column do not meet the peak criteria and are therefore excluded.

We set the relative threshold to η=0.4. Under this setting, all of the detected candidate peaks successfully pass the amplitude filtering condition. For example, 0.30>0.4×0.72=0.288.

(2) Global Statistical Filtering

All candidate peaks from all time frames are merged into a global set $P={⋃}_{l}P_{l}$. We then compute the mean and standard deviation of all magnitude values in the time–frequency matrix M, denoted as $\mu_{M}$ and $\sigma_{M}$, respectively. A global threshold is applied to suppress low-energy or noise-induced peaks. Specifically, only peaks that satisfy:

$$M_{k,l}>\mu_{M}+\beta \cdot \sigma_{M}$$

are retained, where β is a tunable hyperparameter controlling the sensitivity of the threshold.

In this example, the statistical properties of the time–frequency matrix are:

$$\mu_{M}=0.1884,\;\sigma_{M}=0.2396$$

With β=0.3, the resulting global threshold is as follows:

$$\mu_{M}+\beta \cdot \sigma_{M}=0.1884+0.3\times 0.2396=0.2603$$

Therefore, only local peaks with a magnitude greater than **0.2603** are included in the final peak set.

### Appendix A.3. Peak Clustering via DBSCAN

The set of all detected peaks is denoted as $P={⋃}_{l}P_{l}$, where each element is represented as a pair $\left(k,M_{k,l}\right)$ indicating its frequency index and magnitude. To group peaks with similar frequency and amplitude characteristics, we apply the DBSCAN clustering algorithm with a radius of ϵ=0.3 and a frequency–magnitude weighting factor α.

Before clustering, both the frequency indices and magnitudes are independently normalized to the range [0,1] using Z-Score Standardization.(Of course, other normalization methods can be chosen, such as min-max normalization.) To balance their relative importance in distance computation, the magnitude dimension is further scaled by a factor of α. In this experiment, we set α=1.0.

The original peak set *P* contains the following 12 points:

$$\begin{array}{cc} P=\{ & (2,0.80),\;(2,0.82),\;(2,0.81),\;(2,0.83),\;(2,0.81),\;(2,0.82),\\ & \left(2,0.30),\;(2,0.31),\;(2,0.32),\;(6,0.72),\;(6,0.75),\;(6,0.70)\right\} \end{array}$$

After normalization, the transformed feature vectors used for clustering are:

(−0.577,−0.641),(−0.577,−0.737),(−0.577,−0.689),(−0.577,−0.785),(−0.577,−0.689),(−0.577,−0.737),(−0.577,−1.749),(−0.577,−1.701),(−0.577,−1.653),(−1.732,−0.259),(−1.732,−0.402),(−1.732,−0.163)

Applying DBSCAN with ϵ=0.3 and minPts = 1, we obtain the following three clusters:

- $C_{1}=\left\{\left(2,0.80\right),\;\left(2,0.82\right),\;\left(2,0.81\right),\;\left(2,0.83\right),\;\left(2,0.81\right),\;\left(2,0.82\right)\right\}$
- $C_{2}=\left\{\left(2,0.30\right),\;\left(2,0.31\right),\;\left(2,0.32\right)\right\}$
- $C_{3}=\left\{\left(6,0.72\right),\;\left(6,0.75\right),\;\left(6,0.70\right)\right\}$

This clustering result effectively separates peak groups with different energy levels and frequency indices, capturing the underlying structure of the signal.

$$M=\left[\begin{array}{ccccccccc} 0.10 & 0.10 & 0.10 & 0.01 & 0.01 & 0.01 & 0.10 & 0.10 & 0.10\\ 0.30 & 0.32 & 0.33 & 0.11 & 0.10 & 0.12 & 0.32 & 0.31 & 0.33\\ 0.80 & 0.82 & 0.81 & 0.30 & 0.32 & 0.31 & 0.83 & 0.81 & 0.82\\ 0.30 & 0.32 & 0.33 & 0.11 & 0.10 & 0.12 & 0.32 & 0.31 & 0.33\\ 0.10 & 0.10 & 0.10 & 0.01 & 0.01 & 0.01 & 0.10 & 0.10 & 0.10\\ 0.01 & 0.01 & 0.01 & 0.22 & 0.21 & 0.20 & 0.01 & 0.01 & 0.01\\ 0.01 & 0.01 & 0.01 & 0.72 & 0.75 & 0.70 & 0.01 & 0.01 & 0.01\\ 0.01 & 0.01 & 0.01 & 0.22 & 0.21 & 0.20 & 0.01 & 0.01 & 0.01\\ 0.01 & 0.01 & 0.01 & 0.01 & 0.01 & 0.01 & 0.01 & 0.01 & 0.01 \end{array}\right]$$

Different colors in the matrix indicate different clustering results.

### Appendix A.4. Temporal Continuity Processing

We perform temporal continuity processing for each cluster category to capture the temporal consistency of the same class across adjacent time frames. Consider the clustering result:

$$C_{1}=\left\{\left(2,0.80\right),\;\left(2,0.82\right),\;\left(2,0.81\right),\;\left(2,0.83\right),\;\left(2,0.81\right),\;\left(2,0.82\right)\right\}.$$

We associate each peak with its corresponding time index and represent them as ordered triplets $\left(k,M_{k,l},l\right)$. The extended set is as follows:

{(2,0.80,0),(2,0.82,1),(2,0.81,2),(2,0.83,6),(2,0.81,7),(2,0.82,8)}

These points are sorted by time index *l*. To ensure temporal continuity, we define a maximum allowed time gap γ. When the difference between adjacent time indices exceeds γ, the cluster is split accordingly. For example, with γ=1, the set $C_{1}$ is split into two temporally continuous subsets:

- Subset 1: {(2,0.80,0),(2,0.82,1),(2,0.81,2)}
- Subset 2: {(2,0.83,6),(2,0.81,7),(2,0.82,8)}

This process ensures that each region used for interpretation consists of peaks that are not only similar in content but also coherent in time (or temporally continuous).

$$M=\left[\begin{array}{ccccccccc} 0.10 & 0.10 & 0.10 & 0.01 & 0.01 & 0.01 & 0.10 & 0.10 & 0.10\\ 0.30 & 0.32 & 0.33 & 0.11 & 0.10 & 0.12 & 0.32 & 0.31 & 0.33\\ 0.80 & 0.82 & 0.81 & 0.30 & 0.32 & 0.31 & 0.83 & 0.81 & 0.82\\ 0.30 & 0.32 & 0.33 & 0.11 & 0.10 & 0.12 & 0.32 & 0.31 & 0.33\\ 0.10 & 0.10 & 0.10 & 0.01 & 0.01 & 0.01 & 0.10 & 0.10 & 0.10\\ 0.01 & 0.01 & 0.01 & 0.22 & 0.21 & 0.20 & 0.01 & 0.01 & 0.01\\ 0.01 & 0.01 & 0.01 & 0.72 & 0.75 & 0.70 & 0.01 & 0.01 & 0.01\\ 0.01 & 0.01 & 0.01 & 0.22 & 0.21 & 0.20 & 0.01 & 0.01 & 0.01\\ 0.01 & 0.01 & 0.01 & 0.01 & 0.01 & 0.01 & 0.01 & 0.01 & 0.01 \end{array}\right]$$

In the current matrix, the different colors denote what we consider to be distinct homogeneous regions.

### Appendix A.5. Dynamic Boundary Expansion

To address issues caused by spectral leakage, dynamic boundary expansion is applied to capture potentially affected regions. In this example, we perform dynamic boundary expansion on the subset

{(2,0.80,0),(2,0.82,1),(2,0.81,2)},

which corresponds to frequency bin k=2 and time frames l=0 to 2. The magnitude values at this location are [0.80, 0.82, 0.81], yielding a mean energy of 0.81. This value serves as the reference peak energy for subsequent boundary expansion.

The parameters for the boundary expansion are set as follows:

- Energy decay threshold: $\tau_{\mathrm{drop}}=0.25$
- Global energy threshold: $\tau_{\mathrm{global}}=1.0$
- Global mean magnitude of the matrix: $\mu_{M}=0.1884$

Upward expansion (determining $k_{\mathrm{low}}$): We first test the row above (k=1), whose values at frames l=0∼2 are [0.30, 0.32, 0.33]. The average energy is computed as follows:

$${\bar{E}}_{1}=\left(0.30+0.32+0.33\right)/3=0.3167$$

This value is compared with the energy decay threshold:

0.3167>0.25×0.81=0.2025

and the global energy threshold:

$$0.3167>\tau_{\mathrm{global}}\times \mu_{M}=1.0\times 0.1884=0.1884$$

Both conditions are satisfied, so we continue expanding upward.

Next, we test row k=0, with values [0.10, 0.10, 0.10]. Its average energy is:

$${\bar{E}}_{0}=\left(0.10+0.10+0.10\right)/3=0.1000$$

Compared with the decay threshold:

0.1000<0.2025

the condition is not satisfied. Therefore, the expansion stops at k=1, and we set the left frequency boundary to $k_{\mathrm{low}}=1$.

Result: The resulting time–frequency region spans time frames l=0 to 2 and frequency bins k=1 to 2. This region satisfies both temporal continuity and energy-based boundary constraints, and is thus regarded as a valid interpretable unit for downstream analysis.

Note: The same strategy is applied symmetrically to the right-hand side. In this case, the energy values at k=3 also satisfy the boundary conditions, and the right frequency boundary is determined to be $k_{\mathrm{high}}=3$. Therefore, the final frequency boundary is set to [1,3].

$$M=\left[\begin{array}{ccccccccc} 0.10 & 0.10 & 0.10 & 0.01 & 0.01 & 0.01 & 0.10 & 0.10 & 0.10\\ 0.30 & 0.32 & 0.33 & 0.11 & 0.10 & 0.12 & 0.32 & 0.31 & 0.33\\ 0.80 & 0.82 & 0.81 & 0.30 & 0.32 & 0.31 & 0.83 & 0.81 & 0.82\\ 0.30 & 0.32 & 0.33 & 0.11 & 0.10 & 0.12 & 0.32 & 0.31 & 0.33\\ 0.10 & 0.10 & 0.10 & 0.01 & 0.01 & 0.01 & 0.10 & 0.10 & 0.10\\ 0.01 & 0.01 & 0.01 & 0.22 & 0.21 & 0.20 & 0.01 & 0.01 & 0.01\\ 0.01 & 0.01 & 0.01 & 0.72 & 0.75 & 0.70 & 0.01 & 0.01 & 0.01\\ 0.01 & 0.01 & 0.01 & 0.22 & 0.21 & 0.20 & 0.01 & 0.01 & 0.01\\ 0.01 & 0.01 & 0.01 & 0.01 & 0.01 & 0.01 & 0.01 & 0.01 & 0.01 \end{array}\right]$$

In the current matrix, the different colors denote what we consider to be distinct homogeneous regions.

### Appendix A.6. Time–Frequency Block Generation and Optimization

Based on the above procedures, we obtain the following four time–frequency blocks: Given the following four time–frequency blocks:

$$\begin{array}{cc} B_{1} & =(0,2,1,3)\\ B_{2} & =(3,5,2,2)\\ B_{3} & =(6,8,1,3)\\ B_{4} & =(3,5,5,7) \end{array}$$

We compute the pairwise overlap based on both temporal and frequency dimensions. In this example:

- $B_{1}$ and $B_{2}$ have no overlap in the temporal domain, so their overlap is 0.
- $B_{2}$ and $B_{4}$ share the same time range [3,5], but their frequency ranges do not intersect, so the overlap is also 0.

As a result, none of the block pairs exceed the predefined overlap threshold δ=0.3, and no boundary refinement is needed. All blocks are preserved for subsequent analysis.

Note: In practical applications, if two blocks have significant overlap in both time and frequency dimensions (i.e., overlap ≥δ), their boundaries should be adjusted accordingly. This may involve trimming the overlapping area or shifting the block edges while retaining the core structure of each block. To address this, the current algorithm adopts an iterative boundary refinement strategy: at the point of overlap, the boundary indices on both sides are simultaneously reduced, and the degree of intersection is recalculated. This process repeats until the overlap falls below the threshold δ.

## Appendix B. Explanation of Algorithm Parameters

This appendix outlines the key tuning parameters used in our method and the STFT.

### Appendix B.1. STFT Algorithm Parameter Settings

In STFT, the choice of window length *N*, frameshift step *H*, and frequency resolution Δf plays a critical role in the quality of time–frequency analysis. Here, we discuss how to properly configure these parameters.

(1) Window Length *N*

The window length *N* determines the frequency resolution, given by:

(A1) $$\Delta f=\frac{f_{s}}{N}$$

where $f_{s}$ denotes the sampling rate. A larger *N* leads to finer frequency resolution (smaller Δf), which allows better discrimination of adjacent spectral components. However, this comes at the cost of reduced temporal locality, making it harder to detect transient events. Therefore, longer windows are suitable for analyzing stationary or quasi-stationary signals (e.g., vibration, audio), while shorter windows are preferred when analyzing non-stationary signals (e.g., ECG, impulsive noise).

Meanwhile, if researchers prefer not to manually tune this parameter, recent studies offer a promising alternative. Leiber et al. have highlighted the critical role of window length in time–frequency analysis and proposed a differentiable STFT framework, in which the window length is treated as a continuously optimizable parameter. This allows the model to achieve an adaptive trade-off between time and frequency resolution [46].

(2) Frameshift *H*

The hop size *H* determines the temporal spacing between adjacent windows and thus controls the time resolution and the density of the time–frequency representation. Smaller values of *H* yield higher time resolution and smoother spectrograms but also increase computational overhead. A practical range is typically H∈[N/8,N/2], which ensures sufficient temporal continuity while maintaining efficiency.

Meanwhile, if researchers prefer not to manually tune this parameter, recent studies offer potential solutions. Leiber et al. further extended the differentiable STFT framework by treating the hop size as an optimizable parameter, enabling more precise temporal alignment in the learned signal representations [47,48].

(3) Frequency Resolution Trade-off

Improving frequency resolution generally requires increasing the window length, which simultaneously reduces time resolution. Therefore, selecting appropriate values for *N* and *H* must be based on a careful trade-off tailored to the target signal properties. Practical strategies include:

- For low-frequency detection tasks, ensure that $\Delta f<f_{\min}$, where $f_{\min}$ is the smallest frequency component of interest;
- For signals with dynamic variations, select a small *H* so that each change is captured by multiple overlapping frames, avoiding information loss.

In conclusion, the parameters *N* and *H* should not be tuned independently. Instead, they must be jointly considered in the context of the signal’s frequency characteristics, temporal dynamics, and analysis goals to achieve optimal performance.

(4) Window Function $w\left[n\right]$

In STFT, the choice of window function directly affects the trade-off between spectral leakage and frequency resolution. Different window types exhibit different main-lobe widths and side-lobe attenuation characteristics, which influence the accuracy of frequency analysis and the suppression of spectral interference.

Commonly used window functions include the Hamming, Hann, Blackman, and Kaiser windows. Among them, the Hamming and Hann windows offer a good balance between main-lobe width and side-lobe suppression, making them suitable for most time–frequency analysis tasks. The Blackman window provides stronger side-lobe attenuation, which is beneficial for signals with high dynamic range. The Kaiser window allows flexible control over the resolution via a shape parameter, making it suitable for customized analysis needs. Therefore, the selection of the window function should be based on the sparsity of the signal’s spectrum, the desired frequency resolution, and the tolerance to side-lobe leakage.

### Appendix B.2. TF-LIME Algorithm Parameter Settings

In Section 3.3, we introduced the default settings of the TF-LIME algorithm. Here, we provide a detailed explanation of each parameter’s role and discuss how to adjust them when applying the algorithm to different types of data.

(1) Energy Threshold Factor η

This parameter governs the selection of significant peaks during the peak detection phase. In the magnitude spectrogram $M\in R^{L\times K}$ derived from STFT, for each time frame *l*, a frequency bin *k* is considered a peak only if:

(A2) $$M_{l,k}\geq \eta \cdot \max\left(M_{l,:}\right)$$

Here, η is the energy threshold factor that filters out low-energy components which may correspond to background noise. A higher η leads to stricter selection, retaining only prominent spectral peaks; a lower η increases sensitivity but may introduce spurious detections.

Default and recommended range: The typical range of η is 0.3–0.7. In our experiments, we set η=0.5.

Tuning guidelines:

- For signals with dominant frequency components, consider increasing η (e.g., 0.6–0.7) to improve peak purity;
- For flat-spectrum or weak-signal cases, reducing η (e.g., 0.3–0.4) improves sensitivity.

(2) Noise Suppression Coefficient β

This parameter is used to suppress low-energy noise during the peak detection process. In addition to the relative energy threshold η, a candidate peak $M_{l,k}$ must also satisfy the following global criterion:

(A3) $$M_{l,k}\geq \mu_{M}+\beta \cdot \sigma_{M}$$

where $\mu_{M}$ and $\sigma_{M}$ denote the global mean and standard deviation of the time–frequency matrix M, respectively. This condition ensures that detected peaks are not only locally prominent within a frame, but also globally significant relative to the background distribution.

Default and recommended range:The value of β is typically set between 0.5 and 2.0. In our implementation, we use β=1.5 by default.

Tuning guidelines:

- For signals with strong background noise or low signal-to-noise ratios (SNR), increase β (e.g., 1.5–2.0) to enhance detection robustness;
- For cleaner signals or weaker spectral peaks, reduce β (e.g., 0.5–0.8) to avoid missing informative components.

(3) DBSCAN Clustering Radius ϵ

This parameter controls the neighborhood size in the DBSCAN clustering algorithm. Each peak is represented as a frequency–magnitude pair $\left(k,M_{l,k}\right)$, where both frequency indices and magnitude values are standardized independently using Z-score normalization. This ensures that both dimensions are comparably weighted in distance computation. The neighborhood for clustering is defined as follows:

(A4) $${\left(k_{p}-k_{q}\right)}^{2}+\alpha {\left(A_{p}-A_{q}\right)}^{2}\leq \epsilon^{2}$$

Here, $k_{p}$ and $k_{q}$ denote standardized frequency indices, and $A_{p}$ and $A_{q}$ denote standardized magnitudes. α is a scaling factor that balances the influence of frequency and magnitude differences. ϵ controls the tightness of clustering.

Smaller values of ϵ lead to more fine-grained clusters, effectively distinguishing close peaks, while larger values produce coarser groupings, potentially merging distinct peak features.

Default and recommended range: In the standardized feature space, we recommend setting ϵ within the range of 0.3–0.8. The specific value should be selected based on the density and amplitude variation of spectral peaks in the signal.

Tuning guidelines:

- For signals with dense and finely spaced peaks, use a smaller ϵ (e.g., 0.3–0.4);
- For signals with sparse peak patterns or high amplitude variation, use a larger ϵ (e.g., 0.6–0.8).

Note: TF-LIME uses the frequency index by default as a feature input for clustering. If the time–frequency matrix has low-frequency resolution (i.e., each index covers a wide frequency band), we recommend replacing the index with the actual center frequency corresponding to each bin. This adjustment improves the physical accuracy of frequency-based distance calculations and enhances model generalization.

(4) Scaling Factor α

This parameter balances the relative influence of frequency and magnitude in the distance calculation during peak clustering. Since both frequency indices and magnitude values are independently standardized using Z-score normalization, their scales become comparable.

Given this normalization, we set α=1 by default, assigning equal importance to frequency differences and signal intensity variations. This setting has proven robust in practice and is generally not recommended to be changed.

If needed for specific applications, a value of α<1 increases the emphasis on frequency differences, while α>1 places more weight on magnitude (signal strength) differences.

(5) Maximum Time Gap γ

This parameter limits the temporal range over which peaks can be grouped during clustering. Even if two peak clusters have similar frequency and magnitude characteristics, they will not be merged if their time frame difference exceeds γ. This prevents the erroneous grouping of non-contiguous frequency events.

Default and recommended value: We set γ=1 by default, allowing only peaks in adjacent time frames to be considered part of the same group. This setting is well-suited for high temporal resolution scenarios or applications that require strict time consistency, such as non-stationary signal analysis in ECG.

Tuning guidelines:

- Increase γ (e.g., 2–3) when the signal exhibits strong temporal continuity in its spectral features, or when the TFHS algorithm tends to excessively fragment a single-frequency band across adjacent time frames;
- Keep γ=1 or smaller to enforce high temporal discrimination during clustering.

(6) Energy Decay Constraint $\tau_{\mathrm{drop}}$ and Global Energy Threshold $\tau_{\mathrm{global}}$

These two parameters are used in the dynamic boundary expansion phase to determine the left and right frequency limits for each temporally continuous cluster. The boundaries are extended in both frequency directions using a bidirectional search strategy.

$\tau_{\mathrm{drop}}$ controls the acceptable energy decay at the boundary. It ensures that the boundary frequency does not fall into a region of sharply decreasing energy by requiring that the average energy at the boundary remains above a certain ratio of the average energy of the peak points within the cluster.

$\tau_{\mathrm{global}}$ sets a global energy threshold to exclude low-energy frequencies. This helps guarantee that the identified frequency boundaries are not only locally prominent but also globally significant with respect to the entire time–frequency matrix.

Default and recommended values: $\tau_{\mathrm{drop}}$ is set to 0.6 by default, meaning that the boundary frequency must retain at least 60% of the average peak energy within the current cluster; $\tau_{\mathrm{global}}$ is set to 1.2, requiring the boundary frequency to exceed 1.2 times the global average energy.

Tuning guidelines:

- To enforce sharper boundary constraints, increase $\tau_{\mathrm{drop}}$ to 0.7–0.8;
- For simpler signals or when strict boundary conditions are not required, keep $\tau_{\mathrm{drop}}$ low (e.g., 0.3–0.4) to allow more flexible frequency expansion;
- Increase $\tau_{\mathrm{global}}$ (e.g., to 1.5) to suppress noise and exclude weak frequencies;
- For smooth signals or low SNR scenarios, lowering both parameters can help enhance robustness and tolerance.

(7) Overlap Threshold δ

This parameter is used during the time–frequency block generation and optimization phase. After combining the segmentation results from both the time and frequency dimensions, each resulting region is defined as a time–frequency block $B_{n}=\left(l_{l},l_{r},k_{\mathrm{low}},k_{\mathrm{high}}\right)$.

If two blocks exhibit a significant degree of overlap, the algorithm evaluates whether their overlapping area exceeds the threshold δ. If so, their boundaries are automatically adjusted to reduce the shared region, while preserving the main structure of each block. This helps prevent interference and eliminates unnecessary redundancy between blocks.

Default and recommended value: The default value is δ=0.3, meaning that boundary adjustment is triggered when two blocks share 30% or more of their combined area.

Tuning guidelines:

- Decrease δ (e.g., to 0.1) for finer segmentation and stricter overlap control;
- Increase δ (e.g., to 0.3 or above) when some level of overlap is acceptable or useful for representing continuous signal structures.

(8) Segmentation Window window

This parameter controls the size of the sliding window used to partition residual areas of the time–frequency matrix that are not identified by the TFHS algorithm. It is applied as a post-processing step and is designed to divide these unmarked regions into smaller, regular blocks in order to effectively reduce the dimensionality of the input space for interpretability models such as TF-LIME.

During this process, the defined window is used to tile the remaining regions uniformly. Each resulting sub-block is treated independently and passed to the interpretability module.

Default and recommended value: The default window size is (2,2), which means that unassigned areas are divided into sub-blocks of 2 time frames × 2 frequency bins. This setting balances local information retention with effective dimension reduction for interpretation.

Tuning guidelines:

- Use larger windows (e.g., (3,3) or (4,4)) to reduce the number of interpretable units and compress the representation;
- Use smaller windows (e.g., (2,1) or (1,2)) when local structure preservation is important;
- Use the minimum window (1,1) if extremely fine-grained interpretability is desired, such as per-bin attribution analysis. Note that this significantly increases the computational cost.

(9) Number of Generated Perturbation Samples *u*

This parameter controls the number of perturbed samples generated in the TF-LIME algorithm for producing local explanations. TF-LIME extends the classic LIME framework to the time–frequency domain, where each selected time–frequency block is locally perturbed to create synthetic samples. These samples are passed through the model to collect predictions, which are then used to fit a local linear surrogate model for interpreting the original output.

The value of *u* directly affects the stability of the explanation and the fidelity of the surrogate model, as well as the computational cost of the entire interpretability process.

Default and recommended value: In this work, we set u=500 as the default.

Tuning guidelines:

- If the explanation region is simple and the model response is stable, *u* can be reduced to below 500 to improve efficiency;
- For deep neural models or when the time–frequency blocks exhibit complex nonlinear patterns, *u* should be increased above 500 to enhance surrogate model fitting;
- When setting *u*, it is crucial to consider the dimensionality of the interpretability space—i.e., the total number of blocks to be explained.
